# Alzheimer’s disease diagnosis and classification using deep learning techniques

**DOI:** 10.7717/peerj-cs.1177

**Published:** 2022-12-20

**Authors:** Waleed Al Shehri

**Affiliations:** Department of Computer Science, College of Computer in Al-Lith, Umm Al-Qura University, Makkah, Saudi Arabia

**Keywords:** Alzheimer’s disease, Dementia, DenseNet169, ResNet50, CNN, MRI

## Abstract

Alzheimer’s disease is an incurable neurodegenerative disease that affects brain memory mainly in aged people. Alzheimer’s disease occurs worldwide and mainly affects people aged older than 65 years. Early diagnosis for accurate detection is needed for this disease. Manual diagnosis by health specialists is error prone and time consuming due to the large number of patients presenting with the disease. Various techniques have been applied to the diagnosis and classification of Alzheimer’s disease but there is a need for more accuracy in early diagnosis solutions. The model proposed in this research suggests a deep learning-based solution using DenseNet-169 and ResNet-50 CNN architectures for the diagnosis and classification of Alzheimer’s disease. The proposed model classifies Alzheimer’s disease into Non-Dementia, Very Mild Dementia, Mild Dementia, and Moderate Dementia. The DenseNet-169 architecture outperformed in the training and testing phases. The training and testing accuracy values for DenseNet-169 are 0.977 and 0.8382, while the accuracy values for ResNet-50 were 0.8870 and 0.8192. The proposed model is usable for real-time analysis and classification of Alzheimer’s disease.

## Introduction

Alzheimer’s disease (AD) is a type of incurable brain disease due to neurodegeneration. AD is present worldwide. AD is characterized by β-amyloid (Aβ), which contains extracellular plaques and tau-containing intracellular neurofibrillary tangles (Knopman et al., 2021). Cognitive ability disorder is the major symptom due to AD. This disease is more prevalent in aged people, normally affecting those aged 65 or older; 10% of cases are early onset occurring in people younger than 65. AD also affects language, attention, comprehension, reasoning, and memory. Professionals can take care of patients suffering from this disease’s symptoms. The decline in cognitive ability occurs due to dementia which impacts daily activities. AD is the most common type of dementia, accounting for about two-thirds of the cases due to age factors. In 2020, AD was the seventh leading cause of death in the United States of America. AD has some treatments to improve the symptoms, but there is no proper treatment to recover (Kumar et al., 2018).

AD types are classified as Non-Dementia, Very-Mild Dementia, Mild Dementia, and Moderate Dementia. According to AD analysis, these stages are defined according to other research techniques and are different from the DSM-5 classification of AD (Kumar et al., 2018). Symptoms of AD depend upon the stage of the disease. The most common and very first symptom is short-term memory loss. Also, a language disorder is common in AD patients. AD symptoms normally do not show in the early stages, which is the major barrier to proper treatment. As there is no proper treatment for AD, early diagnosis enables potential treatment to cover the early stages. However, early diagnosis is challenging due to the presentation of minor symptoms and sometimes it is not possible to properly identify the symptoms. Normally, a neuropsychological examination is used for the early diagnosis of AD. Clinicians are responsible for properly analyzing the AD patient, but the manual analysis is tedious and takes time for the number of patients symptomatic for the disease (Salehi, Baglat & Gupta, 2020; Butt et al., 2019).

Medical experts are responsible for AD diagnosis, ideally found in the early stages. However, unfortunately, due to the large volume of data in medical images, and the large number of patients, it is impossible to accurately and quickly analyze them manually. Every clinician or medical expert manually examines a few of the records and provides an analysis based on their experience and knowledge. The risk of improper analysis may cause more problems. There is a need for an automatic solution for the analysis of the large volume of imaging data from patients. Various techniques, such as the Internet of Medical Things (IoMT), clinical treatment in labs using MRI or CT images, machine learning-based systems for analyzing large volume data, and deep learning approaches are playing an important role in the medical domain (Kundaram & Pathak, 2021). MICCAI BRATS challenges also provide solutions using deep learning and machine learning approaches on MRI or CT images (Frozza, Lourenco & De Felice, 2018).

## Background

AD is a fast-growing disease occurring worldwide. It mostly affects the aged population. AD is incurable and is a neurodegenerative disease that mostly affects the brain. People are facing problems associated with AD due to a lack of early diagnosis due to no or minor symptoms in the early stages of the disease. Gaudiuso et al. (2020) proposed a technique using machine learning integrated with Laser-Induced Breakdown Spectroscopy (LIBS). Micro drop plasmas were diagnosed for AD patients and healthy controls (HCs). The classification was also performed using machine learning algorithms. The dataset used for the evaluation of the model had 31 AD patients and 36 HCs. The proposed technique successfully diagnosed late-onset AD, with a better diagnosis for patients greater than 65 years of age. The total classification accuracy between AD and HC was 80%, which indicates that it is better than other approaches.

Bilal et al. (2020) proposed a technique for the accurate diagnosis of AD. This study proposed nanotechnologies to overcome the limitations in treatment for early diagnosis and analysis. The neurodegenerative disease was treated with nanocarriers delivering bio-actives. Using nanocarriers with bio-actives is a very fruitful way to treat neurodegenerative disease compared to common therapies. This review came after studying nanocarriers, nanoparticles, and nanotubes which are helpful for early-stage diagnosis in large volumes of data. This study conducted a few experiments to find an optimum solution for early diagnosis for aged people to overcome AD. The results of this study perform better than the open-source dataset and suggest how to treat patients in the early stages.

DeTure & Dickson (2019) proposed a method for the diagnosis of AD, specifically neuropathological diagnosis for AD. AD is the most common disease and a cause of dementia, affecting memory. This study reviews various ways to find an improved solution for the diagnosis of AD. The major problem identified is how to manage the clinical treatment of many patients, especially when there is no properly defined treatment for the disease. Artificial Intelligence and machine learning also play their role in monitoring a large number of patients in a short time, specifically for the early diagnosis of the disease. It is very difficult to diagnose the disease based on symptoms, which include various effects on memory and conversation, and there is a need to address the problems for early diagnosis. This study gives a solution for diagnosis in early AD stages using machine learning approaches.

Chitradevi & Prabha (2020), proposed a solution for diagnosing AD by analyzing brain sub-regions using deep learning approaches and image processing optimization techniques (Kaggle, 2019). The deep learning algorithms used were Genetic Algorithm, Partial Swarm Optimization, Grey Wolf Optimization, and Cuckoo Search algorithm. Grey Wolf Optimization outperformed and was more stable than the other algorithms. Deep learning algorithms were used as validation tools to classify normal and abnormal AD. Among all the sub-regions of the hippocampus is the important region where the maximum chance of detecting AD is found in the early stages. This study evaluated using an open-source dataset and obtained values for accuracy, sensitivity, and specificity of 95%, 95%, and 94%, respectively. This score shows the proposed study is most correlated with the Mini-Mental State Examination.

AD diagnosis has been performed using different deep learning and machine learning algorithms on the image dataset, whereas Sharma, Vijayvargiya & Kumar (2021) compared a few deep learning techniques, specifically convolutional neural network (CNN) architectures, for AD. This study analyzed eight transfer learning techniques for the four classes of AD: Non-Dementia, Very Mild-Dementia, Mild-Dementia, and Moderate Dementia. Eight transfer learning approaches were tested: DenseNet-169, MobileNet-V2, ResNet-101, Inception-V3, ResNet-50, VGG-16, VGG-19 and Inception ResNet-V2. The highest score for evaluating these techniques was valued for accuracy and precision at 98.0% and 98.1%, respectively, with these being the best scores in the medical domain. This study proves that using these techniques for AD diagnosis is effective. A deep feature-based real-time model was introduced (Khan et al., 2020) to predict the AD stage. This technique used CNN, k-nearest neighbours (KNN), and support vector machine (SVM) to classify the AD stage from the image dataset and obtained outstanding performance with 99.21% accuracy values. That proves the performance of this research study.

The state-of-the-art studies clearly show the needed system for the early diagnosis of Alzheimer’s disease. The major contributions of the proposed model are mentioned.

### Main contribution

This research study focuses on diagnosing and classifying AD using an image dataset. Due to the minor symptoms of AD, there is still a need to address the nominated research problems. Following are the key points where this research contributes to the AD study. This study gives a solution for accurate, timely, early AD diagnosis. This study ignores the cause of the disease, which is unknown except for a very small number of familial cases driven by the genetic mutation. DenseNet169 and ResNe50, two CNN architectures, were used to validate proposed techniques to classify normal or abnormal (AD) images with four different stages of AD. The research methodology used to develop the solution addressing the identified challenges of this study is presented in the next section.

## Materials and Methods

### Research gaps key points

This research study explores various techniques to address the nominated research problems to overcome this global disease. A few points needed to be addressed, with some under examination, as discussed below.

- Research into efficient and accurate early diagnosis of AD is needed to provide early treatment options. Automatic techniques are required to handle the large volume of patients’ medical image data (Bilal et al., 2020).
- The disease’s primary cause is unknown except for the very small number of familial cases driven by genetic mutation (DeTure & Dickson, 2019).
- Currently, there is no disease therapy, and there is a need for a solution for large volumes of image data to treat a large volume of patients (Ulep, Saraon & McLea, 2018).

Various challenges exist (WebMD Challenges), such that MICCAI are working on this disease to find solutions for the early diagnosis of patients, which helps with timely, clinical treatment. The proposed model in this research is based on deep learning approaches using the convolutional neural network (CNN) architectures ResNet-50 and DenseNet-169. The proposed model suggests the possibility of the early diagnosis and classification of AD.

This article is structured as follows: “Background” presents background knowledge relevant to this research; “Materials and Methods” discusses the proposed methodology, including the proposed model; “Proposed Methodology” elaborates the analysis of the experimental results from the used techniques and provides a comparative analysis with the state-of-the-art; and, “Experimental Results and Discussion” presents the conclusions of this research.

## Proposed methodology

**Figure 1:**
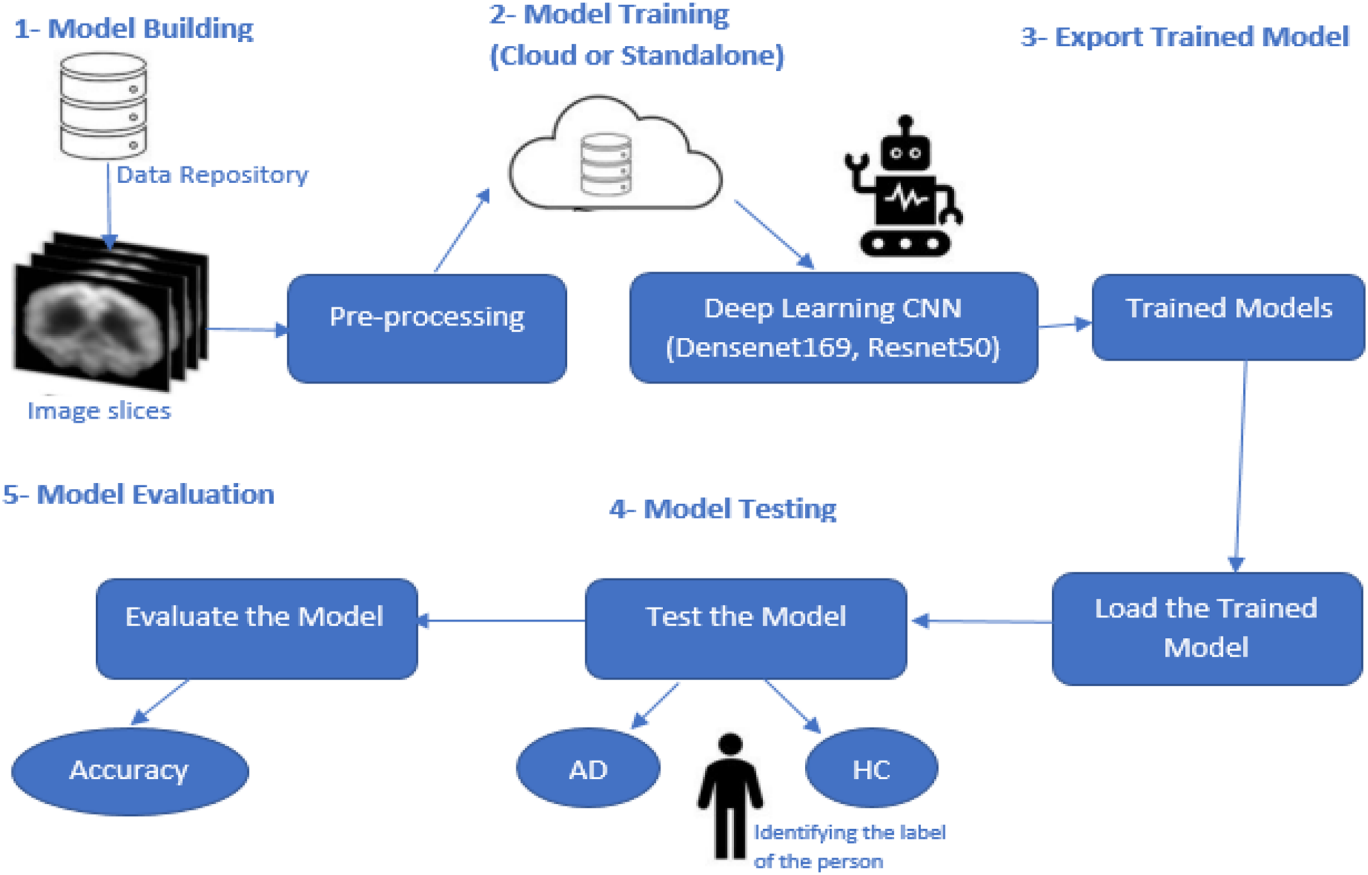
Proposed model methodology.

Figure 1 presents this study’s research methodology for finding a solution to AD’s accurate, early diagnosis. This proposed research methodology addresses the problems discussed in the Introduction. Various techniques based on deep learning were discussed earlier, but these approaches are lacking in the early diagnosis of AD when symptoms are minor or non-existent.

This research study focused on the CNN-based deep learning models known as DenseNet169 and ResNet50, which are used for diagnosing and classifying AD.

### Pre-processing

Several steps are performed in this stage of model development. The first is loading the image dataset into the model. The dataset is insufficient to train the deep learning models to meet the required data volume. Here data augmentation techniques are applied in which a few parameters are set such that the images are rescaled, rotated, zoomed, flipped horizontally and vertically, and split. Validation is performed for the whole image dataset. After applying these steps, sufficient data volume was generated based on the previous dataset. This dataset had four classes: Non-Dementia, Very Mild-Dementia, Mild Dementia, and Moderate Dementia. Each image was individually labelled for analysis purposes. After applying these steps, the dataset was available for further processing.

### Model building

The proposed model was based on the DenseNet169 and ResNet50 CNN models. CNN is a deep learning model used for the classification of features. A CNN model contains several different layers. A few common layers in CNN architectures are discussed below.

#### The input layer

The first CNN layer is the input layer which defines the image size used in the dataset as 240 * 240 * 3 (width * height * channels). Shuffling the images is unnecessary because each training epoch will be shuffled automatically.

#### The convolutional layer

The convolutional layer is the core of the CNN architecture. It is the basic master layer which contains required parameters and feature maps. These layers are key to performance *via* the selection of a kernel. Padding is used in the feature maps and the convolutional layer to match the sizes of the input and output layers. By default, padding is assigned pre-set values of one.

#### Batch normalization layer

Network training is a time-consuming process. Batch normalization of the training data is an easy optimization. Gradients are normalized and help move forward or trigger the network propagation. Batch normalization layers are used between the non-linearity and convolutional layers.

#### ReLU layer

Rectified linear unit (ReLU) is the activation function mostly used in neural network architectures. ReLU layer is used after the batch normalization layer.

#### Max-pooling layer

Spatial features are large. This layer helps to reduce the feature map size and to remove redundancy from the spatial information. Sample reduction helps to reduce the computation cost and to move meaningful information into the feature maps.

#### Fully connected layer

These layers are described by their name. All the previous layers are connected here. At this stage, all the previous learning layers are merged. The final layer provides the classification of the model. The number of outputs equals the number of classes identified in the image dataset. In this study, there are four classes.

#### Softmax layer

The output obtained from the fully connected layer is not normalized. Normalization is performed using an output Softmax layer. The output obtained from this layer is a positive integer and can be used for classification.

#### Classification layer

The classification layer is the last in the CNN architecture. The Softmax layer uses it for classification. Probabilities are returned for each input image to authenticate the manually labelled classes. It also calculates the loss values.

**Figure 2:**
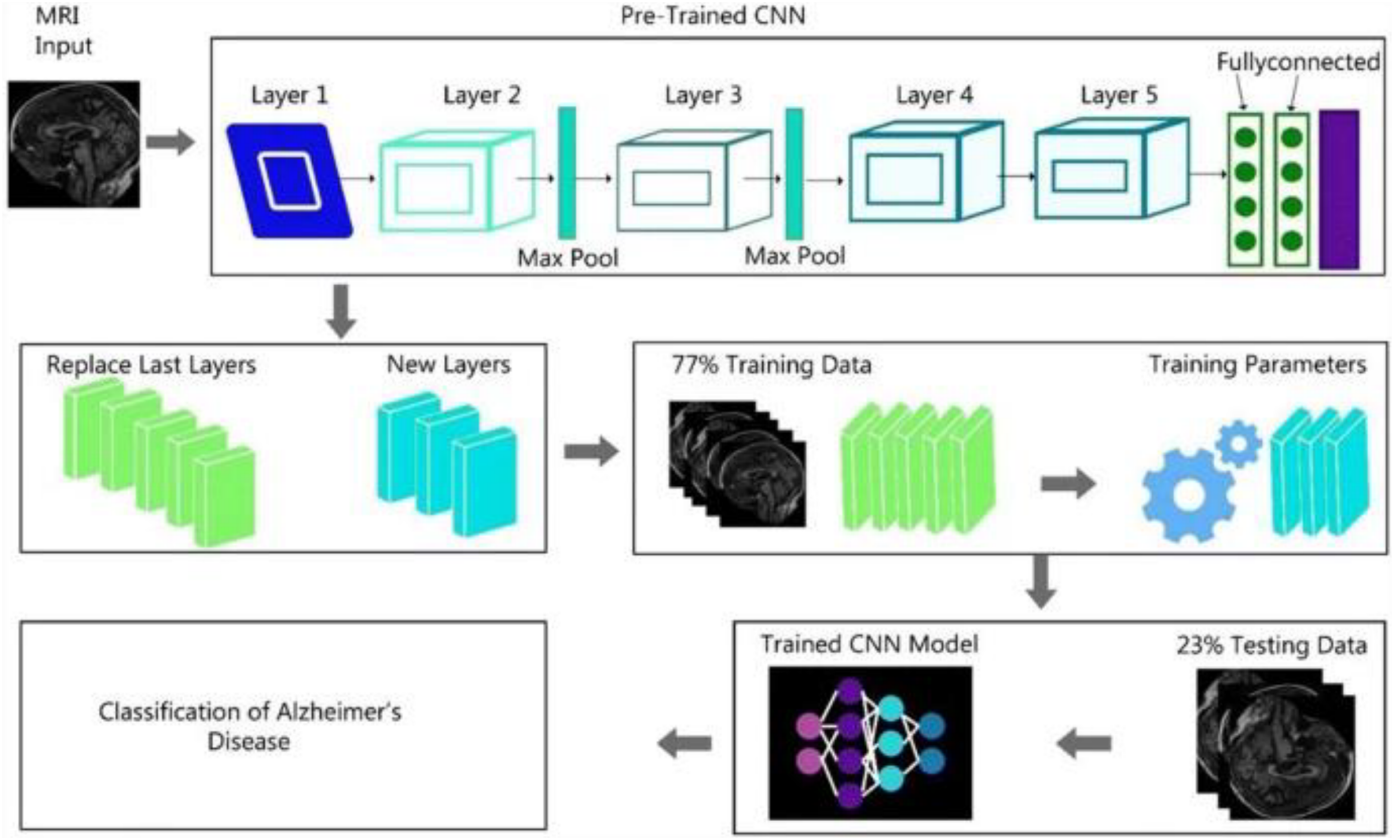
CNN algorithm architecture.

Figure 2 shows the complete generalized architecture for the CNN architecture. In this study, we are using CNN architectures DenseNet169 and ResNet50. The DenseNet169 architecture is based on the CNN architecture divided into various dense blocks.

We discussed that the DenseNet-169 and ResNet-50 are based on the complete CNN architecture. Because these algorithms are the updated versions of the CNN architecture, some modification is performed in these two algorithms, specifically in object detection from images.

A dense convolutional network (DenseNet-169) is a collection of DenseBlocks with many layers. Densenet1-69 has [6, 12, 32, 32] layered structures, but all the basic structures are the same as the CNN architecture. It has a different layered structure. DenseNet-169 can object detection and diagnosis. Another best feature of the DenseNet-169 is the ability to train the dataset more efficiently and use shorter connections between the layers (Nawaz et al., 2021).

**Figure 3:**
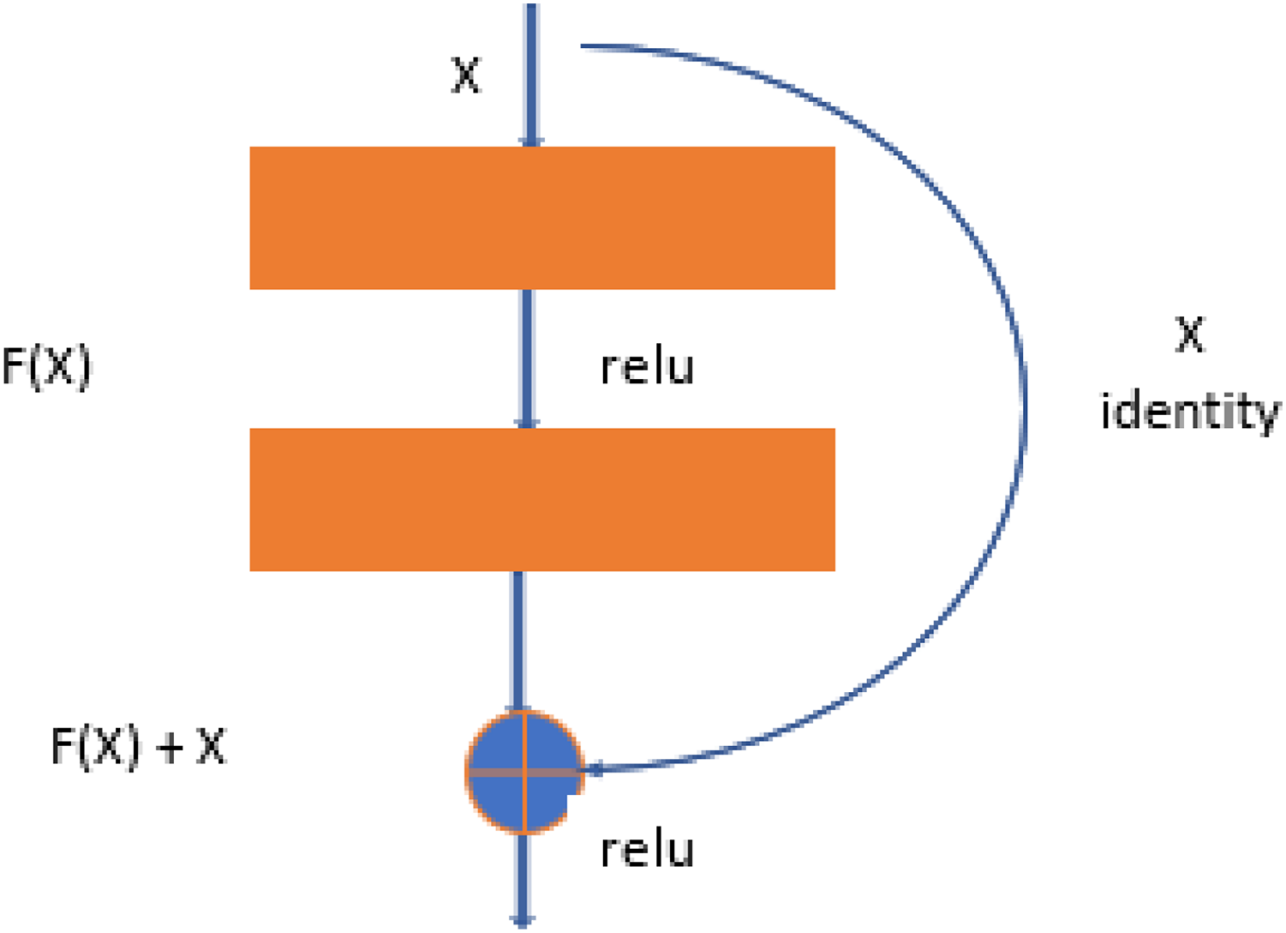
Test case 1 classified the image is healthy brain.

Resnet-50 is also the derived version of CNN architecture, but it is more related to the DenseNet structure. ResNet-50 has a total of 48 convolutional layers. But it has 1 MaxPool layer and 1 Average Pool layer. ResNet-50 has other features, like it has 3.8 × 10^9^ operations of floating. ResNet-50 is the best fit to train the dataset for object detection and diagnosis in the images (Budhiraja & Garg, 2021). The ResNet-50 learning block is shown in Fig. 3.

### Model training

After successfully developing the deep learning model architecture, it needs to be trained. The whole dataset is shuffled in each training epoch for 100. In this study, both architectures, DenseNet169 and ResNet50, were used separately for training purposes. We used 70% of the data for training the model and the rest of the 30% was used to test the model.

### Export-trained model

After training, the trained models were exported for testing and further use. Once the whole dataset has been used to train, there is no need to train again and again. The trained models are used for further processing.

### Testing model

The trained model is tested using various images. This model diagnoses AD and classification between these four different classes. The values output from the model are compared with the true values for the input images. The comparison of output values with true values for the testing dataset is used to evaluate the model.

### Model evaluation

The model is evaluated based on the training model and testing dataset. The evaluation measure is computed by comparing calculated values from the model and true values known for each image in the testing dataset. The evaluation measures are used as proof of whether the model is performing well or not. Accuracy is the evaluation measure used to check the model’s significance. Accuracy is used for the performance analysis of the proposed model. The computation of accuracy is defined in Eq. (1).

(1) $${\rm Accuracy = Aa/Ac *100}$$

where Aa is the number of accurately classified results and Ac is the total number of results.

## Experimental results and discussion

This section elaborates on the dataset used for training and testing the model, the experimental analysis for the proposed model, and the outcomes.

### Dataset

The proposed model was evaluated using an open-source dataset from the Kaggle platform (Fulton et al., 2019). The dataset was collected from various sources and manually labelled with the help of a radiologist. This dataset contains magnetic resonance imaging (MRI) scans. Each image in the dataset is classified into one of four classes: Non-Dementia, Very Mild-Dementia, Mild Dementia, and Moderate Dementia. These image classifications are used for training and testing purposes. The data are separated into two main folders, one for training and the second for testing. The training folder contained 4,098 images, with 1,023 images used for validation. The test folder contained 1,279 images. The whole dataset was used for training and testing purposes with a 70–30% ratio.

### Results using DenseNet-169 and ResNet-50

Table 1 shows the statistical results of the training and testing after the successful execution of the model. DenseNet169 returned better values in the training and testing of the model in comparison with the ResNet50 architecture. Based on this, we decided to use the DenseNet-169 model for further processing. Results and visuals are shown in Fig. 3 with the model loss and model AUC values 10.

**Table 1:** Evaluation of training and testing model.

No.	Algorithm	Training AUC	Testing AUC	Model loss
1	DenseNet-169	0.9777	0.8870	0.3425
2	ResNet-50	0.8382	0.8198	0.9301

**Figure 4:**
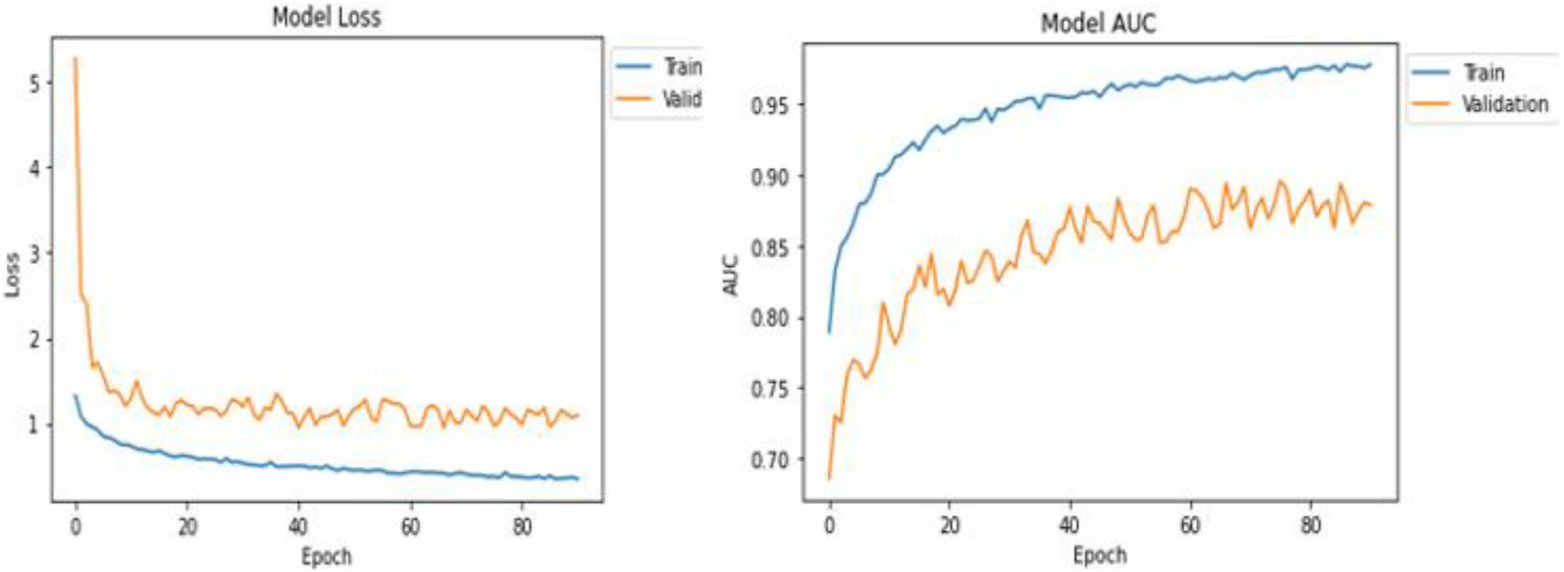
ResNet-50 learning block.

The training results can be seen in Fig. 3 for the DenseNet169 architecture in the form of model loss and model AUC graphs. For the training and validation, DensNet-169 outperforms, as shown in Fig. 4.

**Figure 5:**
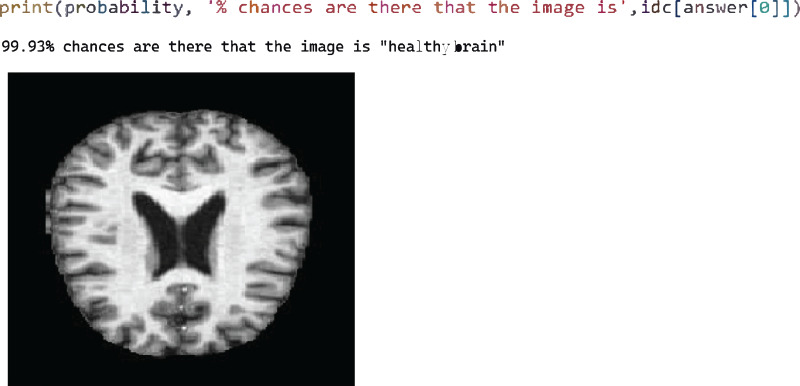
Test case 4 classified the image as very mild AD.

After successfully training the proposed model using the training dataset, the model was evaluated in the test phase, where four test cases were performed sequentially. Figure 5 shows test case 1, where the model successfully classified an image labelled “healthy brain” with a probability of 99.93%. Here we select the trained model and pass the image from the test folder. The classification is the output with the best probability value.

**Figure 6:**
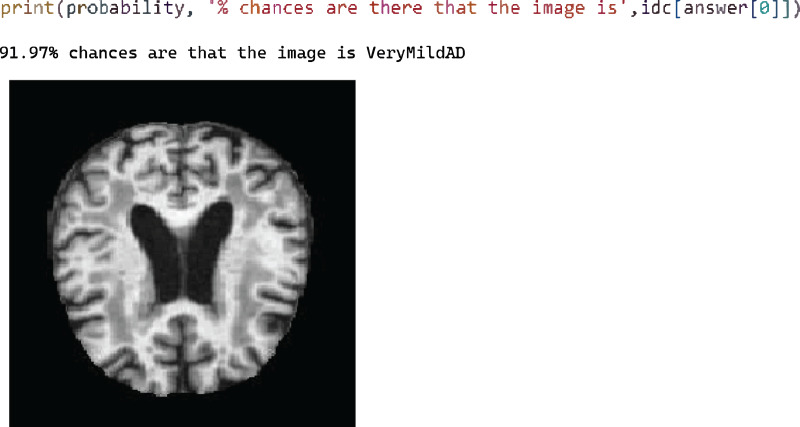
Model loss and AUC representation for the DenseNet169.

Figure 6 shows test case 4, where classification is performed for the “Very Mild AD” class and the proposed model is classified accurately with 91.97% probability.

**Figure 7:**
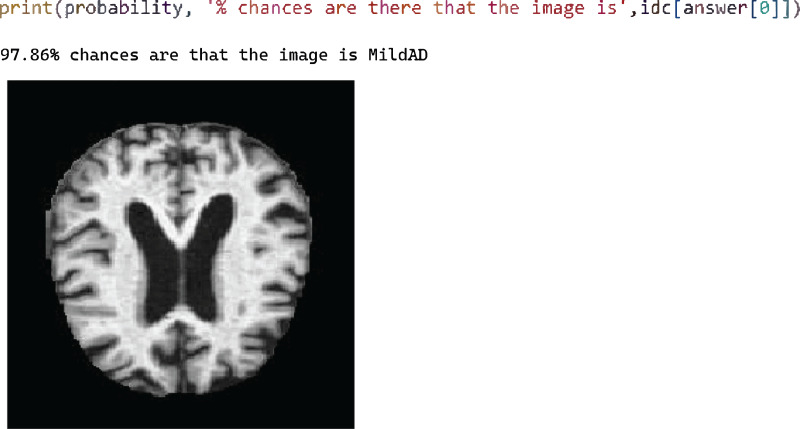
Test case 2 classified the image as mild AD.

Figure 7 shows test case 2 for testing the Mild AD class and the trained model classified it successfully. The probability of the identified classification was 97.86%.

**Figure 8:**
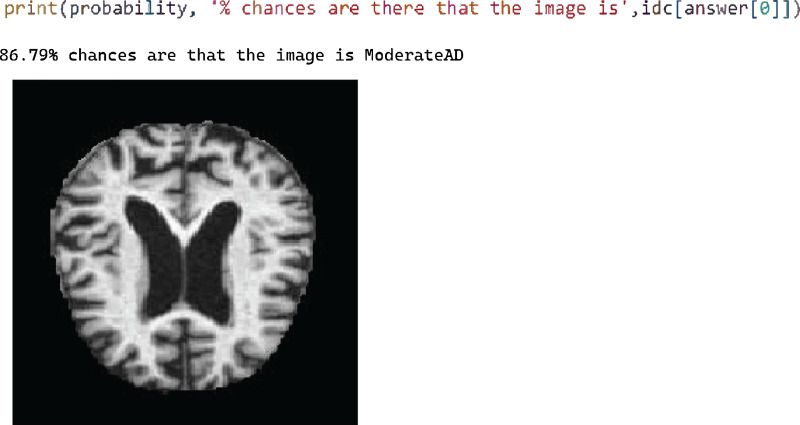
Test case 3 classified the image as moderate AD.

Figure 8 shows the test case 3 results for the Moderate AD test data and the proposed model successfully predicted the classification with 86.79% probability.

After successfully training the proposed model using the DenseNet-169 and ResNet-50 CNN architectures, testing proved that the proposed model outperformed the diagnosis and classification of AD. This application could be used in the future to aid in the real-time diagnosis and classification of medical images.

### Analysis and comparison

The statistical and visual results show that the proposed model outperformed as compared with the other techniques discussed in the literature review. The proposed model gives the solution for accurate early diagnosis and accurate classification. This model can be used for real-time implementation and it will be helpful for the successful classification of Alzheimer’s disease.

## Conclusion and future work

It has been determined that Alzheimer’s disease is an incurable neurodegenerative disease that affects brain memory, particularly in the elderly. Owing to the enormous number of patients, it is impossible to perform manual diagnosis efficiently and health specialists make errors during evaluation due to time constraints and the difficulty of the process. Various procedures are used to diagnose and characterize Alzheimer’s, but an accurate and timely diagnostic solution is required. The proposed model suggests a deep learning-based method for diagnosing and classifying Alzheimer’s disease utilizing the DenseNet-169 and ResNet-50 CNN architectures. Non-Dementia, Very Mild-Dementia, Mild Dementia, and Moderate Dementia were the four classifications of Alzheimer’s Disease in this model. During the training and testing stages, the DenseNet-169 method outperformed. This suggested approach may be used to do real-time analysis and classification of Alzheimer’s disease. In the future, we plan to extend the disease detection with more data sets and use the different measures to detect the system’s accuracy.

## Supplemental Information

10.7717/peerj-cs.1177/supp-1 Supplemental Information 1 Magnetic Resonance Imaging (MRI) scans. Each image in the dataset is classified into one of four classes: Non-Dementia, Very Mild-Dementia, Mild Dementia, and Moderate Dementia. These image classifications are used for training and testing purposes.
The open source dataset is available at Kaggle: https://www.kaggle.com/datasets/tourist55/alzheimers-dataset-4-class-of-images.

10.7717/peerj-cs.1177/supp-2 Supplemental Information 2 Code. This model proposed in this research suggests a deep learning-based solution using DenseNet-169 and ResNet-50 CNN architectures for diagnosing and classifying Alzheimer’s disease as shown the attached code files.

## Additional Information and Declarations

### Data Availability

The following information was supplied regarding data availability: The Magnetic Resonance Imaging (MRI) scans are available from Kaggle: https://www.kaggle.com/datasets/tourist55/alzheimers-dataset-4-class-of-images.
